# Bile Duct Adenoma with Oncocytic Features

**DOI:** 10.1155/2014/282010

**Published:** 2014-01-28

**Authors:** E. J. Johannesen, Zihao Wu, Jason-Scott Holly

**Affiliations:** ^1^Departments of Pathology, University of Missouri Hospitals and Clinics, M263 Medical Science Building, One Hospital Drive, Columbia, MO 65212, USA; ^2^Departments of Surgery, University of Missouri Hospitals and Clinics, M263 Medical Science Building, One Hospital Drive, Columbia, MO 65212, USA

## Abstract

Bile duct adenomas are benign bile duct proliferations usually encountered as an incidental finding. Oncocytic bile duct neoplasms are rare and the majority are malignant. A 61-year-old male with a diagnosis of colorectal adenocarcinoma was undergoing surgery when a small white nodule was discovered on the surface of the right lobe of his liver. This lesion was composed of cytologically bland cells arranged in tightly packed glands. These cells were immunopositive for cytokeratin 7, negative for Hep Par 1, contained mucin, and had a Ki67 proliferation index of 8%. The morphology, immunophenotype, presence of mucin, and normal appearing bile ducts, as well as the increased Ki67 proliferation rate, were consistent with a bile duct adenoma with oxyphilic (oncocytic) change. Oncocytic tumors in the liver are rare; the first described in 1992. Only two bile duct adenomas with oncocytic change have been reported and neither of them had reported mucin production or the presence of normal appearing bile ducts within the lesion.

## 1. Introduction

Bile duct adenomas are benign proliferations of intrahepatic bile ducts. These lesions are usually found under the liver capsule, often as an incidental finding during surgery or at autopsy. Oncocytic bile duct neoplasms in the liver are very rare. Of the cases described, most have been cystic neoplasms with the majority being malignant. A bile duct adenoma with oncocytic features is an exceptionally rare lesion. Only two cases have thus far been reported. Here we report a case of a bile duct lesion with oncocytic features that has elements of a bile duct adenoma, as well as oncocytic and mucinous features.

## 2. Case Summary

A 61-year-old male recently diagnosed with colorectal adenocarcinoma presented for resection of his tumor. A CT scan showed the large mass in the descending and sigmoid colon with several small lymph nodes in the mesocolon suspicious for metastatic disease. No abnormalities were identified in the liver, gallbladder, pancreas, or spleen. During surgery, a small white 0.3 cm diameter nodule was noted on the surface of the right lobe of the liver. Thought to be possible metastatic disease, this lesion was excised and sent for pathologic evaluation.

**Figure 1:**
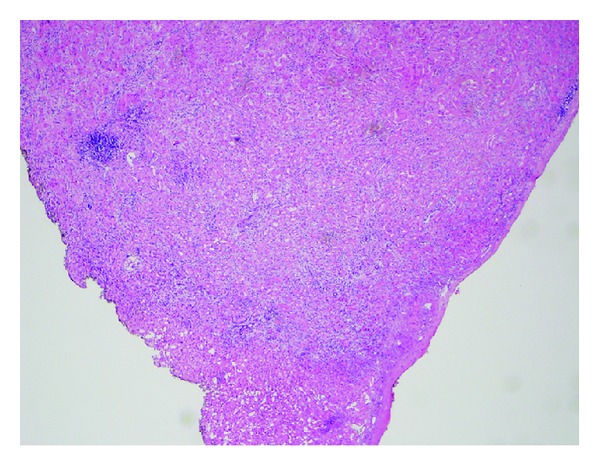
Overview of bile duct adenoma. The well-circumscribed lesion consists of tightly packed glands. H&E.

**Figure 2:**
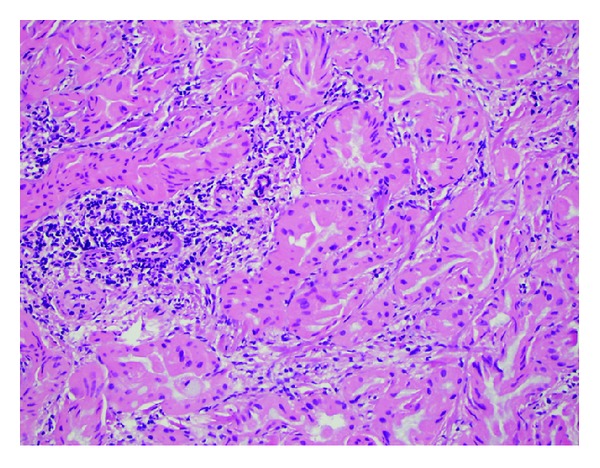
The bile duct adenoma consists of small glands with cells with abundant eosinophilic cytoplasm with small foci of chronic inflammation. H&E.

**Figure 3:**
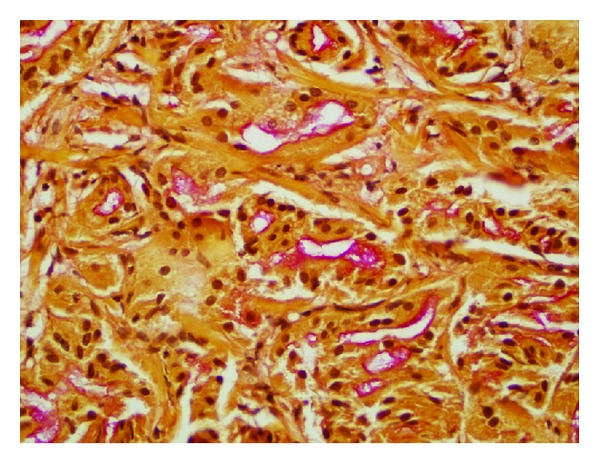
Most of the apical surfaces of the tumor cells and the lumens of the small glands contain mucin, highlighted (red) by this mucicarmine stain.

**Figure 4:**
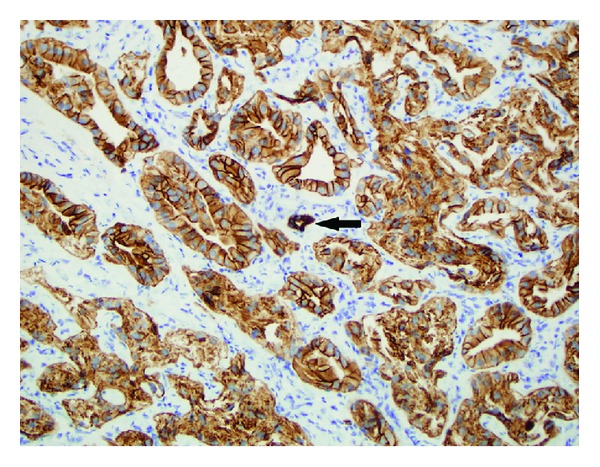
The adenoma cells all have strong cytoplasmic and membranous staining of cytokeratin 7; a normal appearing bile duct is present in the center (black arrow).

**Figure 5:**
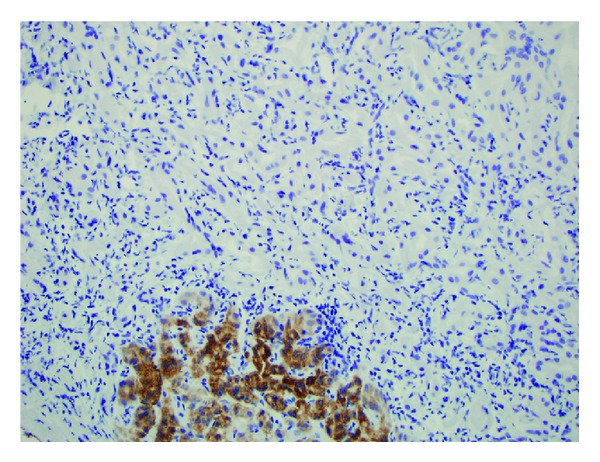
A Hep Par 1 immunostain (Och1E5) marks the cytoplasm of some normal hepatocytes at the edge of the tumor, but the tumor cells are all negative.

Pathologic examination showed a well-circumscribed lesion composed of cytologically bland cells arranged in tightly packed glands with fibrous stroma. There was associated moderate chronic inflammation. The tumor did not invade the adjacent liver (Figure 1). No cystic spaces, bile duct dilation, or inspissated bile was identified. The neoplastic cells had small, round nuclei and abundant eosinophilic cytoplasm, typical for oncocytic cells (Figure 2). Although some anisonucleosis was seen, no significant pleomorphism or mitotic figures were identified. Mucin was noted in some of the lumens, which was confirmed with a mucicarmine stain (Figure 3). Normal bile ducts were also seen within the lesion. No ovarian type stroma was identified. Immunohistochemically, the cells were positive for cytokeratin 7 (CK 7) (Figure 4) and negative for Hep Par1 (Och1E5) (Figure 5), confirming that the cells were biliary in origin. A Ki67 stain showed approximately 8% proliferation. For purposes of comparison, the surgical pathology files at our institution were searched for conventional bile duct adenomas of the liver. Two cases were identified. The blocks were retrieved and a Ki67 stain was performed. One conventional adenoma showed approximately 5% proliferation, while the other showed approximately 2% proliferation. The overall morphologic features and immunophenotype were consistent with a bile duct adenoma with oncocytic change.

The sigmoid colon contained a moderately differentiated adenocarcinoma that invaded through the muscularis propria and into the pericolic fat. The carcinoma was composed of complex glands containing cells with pleomorphic nuclei. Numerous mitoses were present. No lymph node metastasis was identified. The tumor was negative for cytokeratin 7 (CK7). The morphology and immunophenotype were consistent with a primary colonic adenocarcinoma.

## 3. Discussion

Oncocytic tumors in the liver are rare. The first oncocytic lesion was described in 1992 [1]. Although several oncocytic liver, biliary, and pancreatic neoplasms have been described, only two cases of bile duct adenomas with oncocytic change have been reported in the literature. One case occurred in a 57-year-old woman [2] and the other in a 68-year-old man [3]. In both cases, the reports described a proliferation of bile ducts composed of cytologically bland cells with round nuclei. The biliary origin of these cells was confirmed by CK7 positive staining. Neither of the cases reported mucin production, which was present in the current case. All other cases reported thus far have been cystic papillary lesions with the majority being malignant [1, 4–6]. One study classified mucin producing bile duct tumors into columnar type and cuboidal type [7]. All of the cuboidal type tumors that had oncocytic cells were carcinomas. Our case contains elements of both bile duct adenomas and mucin producing neoplasms. Our lesion was small and well circumscribed. It was a solid nodule, not a cystic lesion. There were no papillary formations and no ovarian type stroma. It was positive for CK7 and negative for Hep Par 1, whereas, in two reported cases of papillary cystic oncocytic biliary tumors, the described cells were positive for hepatocyte specific antibodies [4, 5].

In our case, in addition to the mucin production, there were also scattered, normal appearing bile ducts within the lesion. Overall, this is consistent with a bile duct adenoma with oncocytic change. There have been recorded cases of oncocytic metaplasia in other sites, most notably the nasopharynx [8], but not within the biliary system. Bile duct adenomas with oncocytic change are very rare lesions. Only three cases, including this one, have been described to date. Another interesting feature is the slightly increased proliferation index compared to the two conventional bile duct adenomas. For comparison, Shibahara et al. [7] reported an average Ki67 labeling in the cuboidal bile duct carcinomas of 16.6%. In a case report of an oncocytic papillary neoplasm of the liver [5] the Ki67 labeling was 10%. The lower proliferation rate of our lesion is consistent with a benign entity; however, it is higher than the proliferation rate of the two conventional bile duct adenomas. The presence of mucin production is also an interesting feature. Although conventional bile duct adenomas are known to be mucin producing, the two documented cases of oncocytic bile duct adenomas did not mention mucin production. Although this lesion is benign, it is interesting to wonder whether or not this rare entity could be a possible precursor to the oncocytic papillary neoplasms and/or cystadenocarcinomas. The cytological features and mucin production suggest a possible connection, but the positivity of some of the oncocytic neoplasms for hepatocyte specific antibodies could be an argument against this. If a possible connection is to be elucidated, more of these rare entities will have to be studied.
